# How does problem-solving pedagogy affect creativity? A meta-analysis of empirical studies

**DOI:** 10.3389/fpsyg.2024.1287082

**Published:** 2024-02-15

**Authors:** Zehui Zhan, Luyao He, Xuanyan Zhong

**Affiliations:** ^1^School of Information Technology in Education, South China Normal University, Guangzhou, China; ^2^Key Laboratory of Brain, Cognition and Education Sciences, Ministry of Education, Guangzhou, China; ^3^Hua LuoGeng Senior High School, Huizhou, Guangdong, China

**Keywords:** creativity, problem-solving pedagogy, meta-analysis, empirical studies, mixed effects model

## Abstract

The purpose of this study was to investigate the effects of problem-solving pedagogy on individual students’ creativity in different teaching contexts and conditions, and to examine the role of moderating variables that may affect the overall effect size. The study screened 19 relevant studies from the Web of Science for inclusion in the meta-analysis, and extracted 77 effect sizes from these studies that could be used in the meta-analysis. The study first explored the relationship between problem-solving pedagogy and the development of learner creativity, and further analysis focused on moderating variables to investigate the effects of instructional method, grouping method, grade level, problem-solving scaffolding, source of problems, the application of thinking tools, and the application of technology. The results showed that problem-solving pedagogy enhances students’ creativity, while at the same time, among the sources of problems, student-discovered problems are most conducive to creativity stimulation, while teacher-determined problems and problems that lead to student discovery are less effective in terms of promoting creativity. Among the grouping methods, heterogeneous grouping can better utilize the creativity cultivation effect of the problem-solving pedagogy than random grouping and homogeneous grouping. Among the different levels of grades, learners in elementary school are better able to gain creativity under the influence of problem-solving pedagogy than learners in middle school, high school, college, and those in on-the-job learning. However, this study did not reveal any significant benefits for creativity enhancement under the four conditions of instructional method, problem-solving scaffolding, thinking tools-assisted, or technology. The results of this study clarify the importance of problem-solving pedagogy for creativity development, and also reveal the actual effects of the various ways of applying problem-solving pedagogy on creativity development as well as the problems that exist, which provides inspiration for how to better utilize problem-solving pedagogy in the future.

## 1 Introduction

Nowadays, human beings are facing many complex global problems involving the environment and ecology, society and culture, science and technology, and so on. In order to cope with these changes in the new era, countries, regions and organizations all over the world have carefully considered the cultivation of innovative talents in line with the needs of the era’s development. From 1996 to now, UNESCO, the European Union, the United States, Singapore, South Korea, Japan, China and many other countries have put forward national core literacy frameworks in line with their own national conditions. Scholars such as Shi et al. (2016) have summarized the frameworks and elements of various 21st-century core literacies, and found that creativity and problem-solving abilities are the common points of concern for many countries, regions, and organizations. Creativity, on the other hand, is a combination of both creativity and problem-solving; that is, it is the ability to propose or generate multiple novel, original, and appropriate solutions to problems (Atkinson, 2000; Carbonell-Carrera et al., 2019), and is a critical skill for students to survive and thrive in the 21st century (Griffin and Care, 2014).

In response to the above mentioned need for creativity, many studies have conducted in-depth research on how to cultivate creativity. For example, Weng et al. (2022) combined the characteristics of the 5E (engage, explore, explain, elaborate, and evaluate) learning cycle, based on which they designed a creativity project integrating real-world problems to cultivate students’ creativity, while Fan and Cai (2022) analyzed the mechanism of the learning environment’s role in learners’ creativity development. The findings suggest that creative learning environments can significantly enhance students’ learning goal orientation, networking, and knowledge sharing, thus promoting their creativity.

Moreover, in these studies, the most common approach is problem-solving pedagogy (PSP), which is different from Problem-Based Learning (PBL) in that, although both are based on a problem, the goal of PBL is to allow learners to actively construct new knowledge on the basis of existing knowledge (Awang and Ramly, 2008; Albanese and Dast, 2013). It is characterized by two main features: 1. Learners are involved in problem-solving and problem-solving tasks based on course content. 2. Students are required to solve problems completely or partially independently (Rashidov, 2022). In contrast, PSP aims to improve students’ thinking skills, and the acquisition of knowledge is only one of the ways in which they can solve problems; students can solve problems independently or collaboratively, but they must come up with a complete and feasible solution to the problem. Teachers can provide students with a variety of scaffolding or technology support during this process to facilitate thinking activities during problem-solving.

The PSP tends to better promote student creativity, partly because creativity refers to the freedom of an individual to utilize his or her own mind to generate new ideas (Ozkan and Umdu Topsakal, 2021), a process that often requires the integration of cross-disciplinary content as well as diverse perspectives. PSP is not limited to disciplinary content, but rather to the integration of knowledge and perspectives from different domains by providing complex problem situations and encouraging students to come up with as many ideas as possible. They can use a variety of creative techniques or tools in the process to solve problems in new and original ways. On the other hand, because it aims at competence, and creativity is something that needs to be fostered through entrepreneurial practices in the form of services, solutions, or products (Del Campo, 2017), Zhan et al. (2022c) also surfaces that product-based pedagogy promotes creativity. PSP also tends to design its pedagogical tasks around this problem-solving aspect rather than the variation of knowledge, and requires learners to validate the solutions to the problems they propose. For example, in order to promote creativity training, Wang et al. (2019) constructed a problem-solving process model that includes six segments: Mess Finding, Data Finding, Problem Finding, Idea Finding, Solution Finding, and Acceptance Finding, where each segment corresponds to a different task in the classroom. The teacher releases the creative writing task to students by introducing a scenario, and students define the problem according to the task proposed by the teacher, find out the goal (Mess Finding), and complete a handout (Data Finding) according to the goal by searching for relevant resources and cases on the Internet. Subsequently, students further reflect on the usability of the resources they have collected (problem discovery), and based on the available information in one group, they will conduct creative inquiry (Creative Discovery) and modification of the creative outcome (Solution Discovery) to arrive at the final success and discuss the plan for the subsequent presentation of the outcome (Acceptance Discovery). Generally, PSP is a development of PBL, which is usually characterized by competency centeredness, multidisciplinary integration, and implementation of creative solutions.

However, education has the dual role of fostering creativity and stifling it (Burleson, 2005). Although it is often assumed that PSP directly corresponds to the development of creativity (Puccio and Cabra, 2010), it appears from the results of empirical studies that the impact of PSP on the development of students’ creativity has not yet been able to be concluded with total certainty. Some scholars have questioned the minimal effect of teaching the creative problem-solving process on creativity enhancement. For example, Klegeris et al. (2013) argued that PSP-guided instruction breaks down tasks into individual problems presented one by one, which leads to difficulties in ensuring the overall effectiveness and transferability of students’ activity skills. It has also been argued that these conflicts should be attributed to the design of specific instructional interventions, for example, the effectiveness of problem-solving pedagogy may differ between collaborative and individual learning approaches. Korde and Paulus (2017) compared the differences in the performance of individuals and groups in problem-solving brainstorming activities, and found that limited-sized groups outperformed independent individuals. However, Chen and Agrawal (2017) showed that communication hindrance problems associated with team-based learning significantly interfere with creativity development. The conflicting results of these studies make it difficult for educators to decide whether or not they should use creative problem-solving theories and tools for teaching, and to make decisions on the question of how to organize creative problem-solving instruction well. Therefore, in the view of this study, it is essential to understand the key pedagogical conditions under which PSP has a positive effect on creativity.

Prior to this study, there have been systematic literature reviews and meta-analyzes discussing the influencing elements of developing students’ creativity. The focus of these studies has varied. In terms of systematic literature review studies, Reiter-Palmon and Murugavel (2018) summarized the favorable and unfavorable factors affecting the three creative processes of problem construction, idea generation, and idea evaluation to provide ideas for pedagogical research on facilitating creativity development. Sharmin (2021), on the other hand, delved deeper into creativity in computer science education, and the effect of enhancement strategies on students’ learning experiences by reviewing previous literature and extrapolating six core components of creativity enhancement activities, namely: collaboration, relevance, autonomy, ownership, hands-on learning, and visual feedback. Aguilera and Ortiz-Revilla (2021) conducted a systematic search of papers from 2010 to 2020 to summarize 14 instructional interventions on creativity, and conducted an in-depth analysis to explore the impact of both STEAM and STEM teaching approaches on students’ creativity. Similarly, a qualitative research methodology was used to outline what instructional conditions have been researched to support collaborative creativity or creative collaboration (Barrett et al., 2021). However, the study was limited to music education and both of the abovementioned systematic literature reviews lacked the support of quantitative data and discussion of effect sizes. In terms of meta-analysis, Pacauskas and Rajala (2017) used a meta-analytic approach to analyze 24 relevant studies to explore the effects of factors such as users’ interest in performing a task, whether or not they are in a state of mind-flow, and cognitive load on their creative performance.

In summary, previous studies have provided insightful conclusions about developing students’ creativity. However, the aforementioned studies also have some limitations. First, some of the studies focused only on specific disciplines, rather than taking a general creativity perspective. Second, most of the studies failed to explore the effects of specific teaching methods on creativity development in a more systematic way, remaining more at the level of fragmented elements. It follows that the question about what kind of creative problem-solving instructional design is conducive to developing students’ creativity deserves a complementary response in this study. Therefore, the main purpose of this study was to examine the effectiveness of PSP on students’ creativity in different instructional contexts and conditions, and to test the role of moderating variables that may affect the overall effect size. The meta-analysis in this paper was designed to respond to the following nine research questions:

(1) What is the overall effect of PSP on individual student creativity? The reason for proposing this question is because the overall effect of PSP on individual students’ creativity is still controversial nowadays (Puccio and Cabra, 2010; Klegeris et al., 2013). A synergistic and comprehensive final conclusion is needed for future educational practice.
(2) Are there differences in the specific effects of PSP on creativity in terms of fluency, flexibility, and originality? The reason for proposing this question is because creativity is usually assessed in three dimensions (i.e., fluency, flexibility, and originality) (Zhan et al., 2022a,b). It would be interesting to see whether PSP is equally important to all the three dimensions, or is it specifically significant to one of them.
(3) Does instructional method (collaborative group vs. individual learning) affect the overall effect under the conditions of PSP application? The reason for proposing this question is because there is tremendous discussion on the different effect of PSP on students’ creativity between collaborative and individual learning approaches (Chen and Agrawal, 2017; Korde and Paulus, 2017). It will be beneficial to find out the answer through a meta-analysis with effect size of a collection of empirical studies.
(4) Does grouping method affect students’ creativity development under the conditions of PSP application? The reason for proposing this question is because group composition affects students’ creativity and performance to a large degree through peer interaction. For example, Han et al. (2013) study enumerated three methods of random grouping, homogeneous grouping and heterogeneous grouping, and found through empirical experiments that the creativity improvement of students in random grouping was significantly higher than that in homogeneous grouping and heterogeneous grouping. King et al. (1998) also claimed that group members with the same level of ability may provide better feedback for other group members to think and learn. However, if students are in different positions or group members have different abilities, they are less likely to interact effectively and often encounter difficulty on higher-level communication (Vincent and Ley, 1999).
(5) Does learners’ grade level (i.e., elementary school, middle school, etc.) affect the effectiveness of PSP in terms of fostering students’ creativity? The reason for proposing this question is because previous studies revealed that students in different grade level represent different creativity status when adopting PSP treatments. For example, students in the high school level showed no significant difference according to their grade level and gender (Le et al., 2022), while students in university level was highly affected by the treatment (Wu et al., 2014). Therefore, grade level might be a potential regulated variable among the studies, which needs to be taken into consideration.
(6) In terms of the problem-solving process, does the presence or absence of problem-solving scaffolding support affect the effectiveness of PSP in terms of fostering students’ creativity? The reason for proposing this question is because problem-solving scaffolding is commonly used as important strategies in PSP. During the PSP process, scaffolding could be a good promoter to trigger students’ creativity, while it could also be a boundary of hindering students’ creativity by step-by-step regulation. Therefore, it is taken into consideration in the meta-analysis.
(7) In terms of the way of posing problems, do different sources of problems affect the effectiveness of PSP in terms of fostering students’ creativity? The reason for proposing this question is because creativity originated by the initial problem discovery, and all breakthroughs in science and technology are often the result of an obsession with problems. Generally, learning often begins with a focus on a problem, followed by mastery of the problem-solving method and the use of multifaceted thinking (Tan et al., 2009). However, there is opposite view on the effect of problem sources. For example, Bonotto and Santo (2015) believed that problem raising is a form of creative activity, and allowing learners to raise their own questions is best for developing their critical thinking and creativity. On the contrary, Kim et al. (2014) believed that the development of students’ creativity should be led by teachers, and proposing driving questions is one of the most important treatments for guidance, so questions posed should be well-designed by teachers and proposed in suitable situations that help students link previous and new knowledge. Therefore, the source of problem is also counted in this meta-analysis.
(8) In terms of the application of thinking tools, does the presence or absence of the support of thinking tools affect the effectiveness of PSP in terms of fostering students’ creativity? The reason for proposing this question is because creativity is accompanied by a series of thinking activities, thus thinking tools (e.g., brainstorming, six thinking hats, empathy map, etc.) might help to support the development of students’ creativity when adopting PSP. That’s why the condition of thinking-tools assisted is also taking into consideration.
(9) In terms of information technology intervention, does the presence or absence of information technology support affect the effectiveness of PSP in terms of fostering students’ creativity? The reason for proposing this question is because technology has been commonly used in education, and there were controversial statements on the impact of different aspects of technology on creativity. For example, Kim et al. (2014) explored the influence of technology on creativity in intelligent education, and pointed out that four technical factors (i.e., technology self-efficacy, collaboration, resource, interactivity) were the main factors affecting students’ creativity (Xu et al., 2023). While technology is a double-edged sword. Another study found that the overuse of information technology may lead to greater stress and reduce creativity (Oldhaml and Da Silva, 2015). Therefore, the exploration on the application of technology in PSP is necessary.

## 2 Method

### 2.1 Data sources and search strategy

This study used a keyword search method to retrieve journal articles and systematic literature reviews from different databases, the sources of which include the Web of Science, CNKI, ProQuest, Science Direct, and Springer. First, we jointly conducted an initial search using possible combinations of the following three sets of keywords: set A = {creativity, creative}, set B = {problem-solving}, and set C = {teaching, education, instruction}. Search terms included, but were not limited to, “creative problem-solving” AND education, creative AND “problem-solving” AND education, creative* AND “problem-solving.”

The literature search was centered on the Web of Science and CNKI databases, supplemented by ProQuest, Science Direct, and Springer databases, and restricted to academic journals and conference papers as the form of literature publication. In addition, original studies should meet the following criteria to be included in this meta-analysis, and the specific inclusion and exclusion rules are shown in Table 1.

**Table 1:** The inclusion and exclusion criteria.

Criteria	
Inclusion	Studies in which creativity is the primary dependent variable measured and results are reported for specific variables such as fluency, flexibility, and originality
	Peer-reviewed studies
	Studies in which the topic is related to creativity, and teaching creative problem-solving
	Studies that utilize a single-group pre- and post-test experimental or quasi-experimental research methodology and report data (e.g., sample sizes, means, standard deviations, etc.) that can be used to calculate effect sizes
Exclusion	The study was published before 2000
	Studies were written in languages other than English or Chinese
	Studies that focused on cognitive outcomes (e.g., beliefs, attitudes, and motivation, etc.) and personality (e.g., risk-taking, curiosity, etc.) in creativity, as measured by self-reported surveys, were excluded
	Studies that did not include sufficient statistical information to calculate correlation coefficients between creative problem-solving and creativity

**Figure 1:**
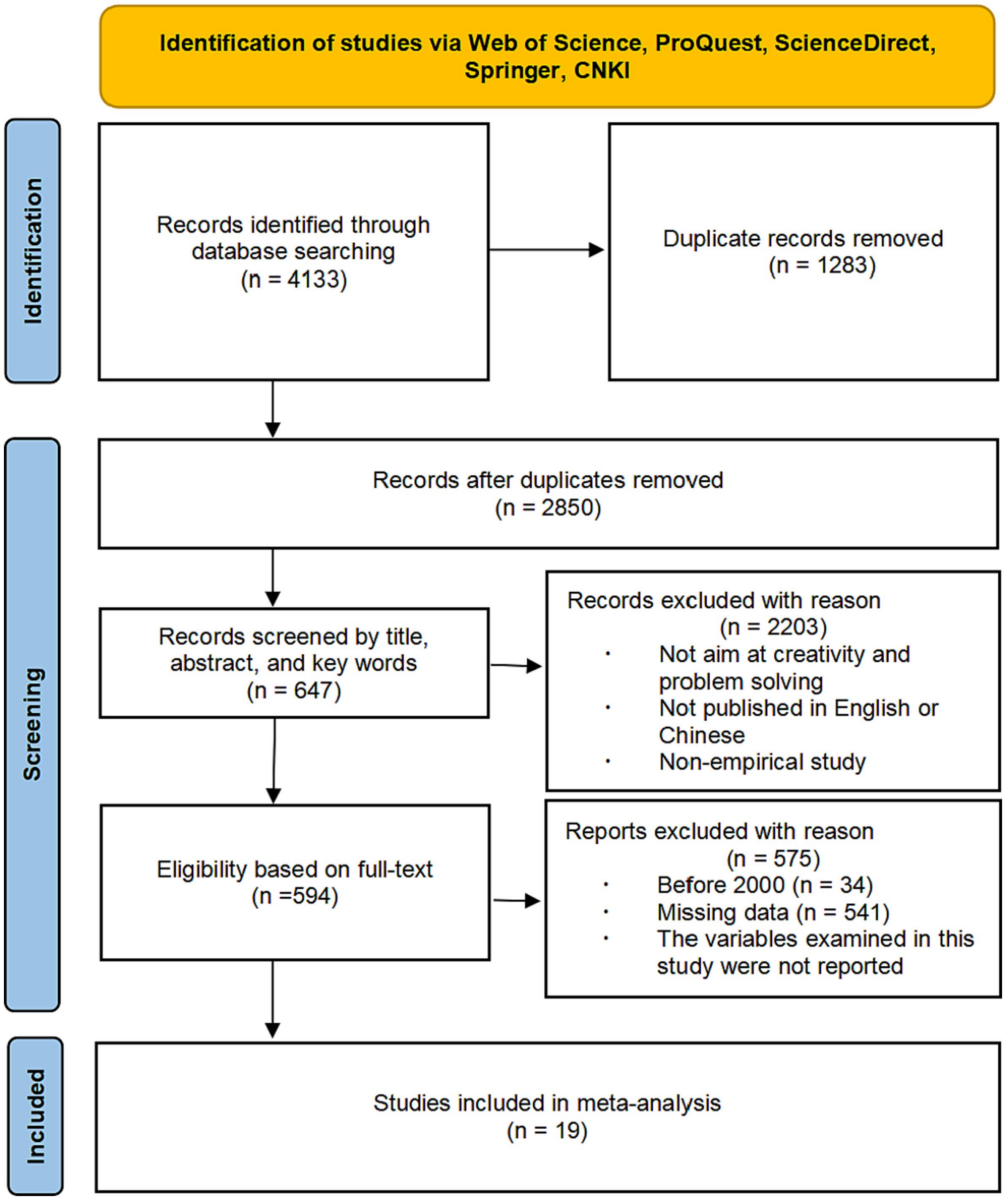
The procedure of inclusion and exclusion of studies for the keyword search.

The search record for this study was updated to January 5, 2023, with a total of 4,133 studies retrieved, and the literature search was executed according to the PRISMA process (Page et al., 2021), which ultimately resulted in 19 studies being screened for inclusion in the meta-analysis by the two researchers, which yielded a total of 77 effect sizes that could be used in the meta-analysis. The specific search process is shown in Figure 1.

### 2.2 Eigenvalue encoding

To address the eight issues of concern, this study coded aspects of pedagogy, collaborative process and scaffolding tools in creative problem-solving based on previous literature. The specific content of the coding table includes basic information about the study (author, year of publication, country), the study population (total number of participants, number of participants in the experimental and control groups, and grade level), the experimental intervention (information about moderating variables such as grouping methods, instructional methods and content, and the application of information technology (IT) tools and other pedagogical aids), and the measurement tools and results (mean and standard deviation of the experimental and control groups, or other statistical data from which effect sizes can be calculated), as shown in Table 2. The classification coding process was done independently by two researchers, and the coding consistency coefficient Cohen’s Kappa value was 0.95, which indicated a satisfactory coding reliability.

**Table 2:** The coding table adopted in this study.

Categories	Codes
Grade level	Kindergarten (K), Elementary School (E), Middle School (M), Higher School (H), University (U), Others (O)
Instructional method	Individual Problem-solving (IPS), Collaborative Problem-solving (CPS)
Grouping method	Heterogeneous Grouping (HeG), Homogeneous Grouping (HoG), Random Grouping (RG), Students Grouping (SG)
Sources of problem	teacher-prescribed questions (T), Instructional material-guided questions (I), and student-discovered questions (S)
Problem-solving scaffolding	No, Yes
Application of thinking tools	No, Yes
Application of technology	No, Yes

#### 2.2.1 Coding

##### 2.2.1.1 Sources of problems

This study focuses attention on the sources of questions, which can be categorized into three types, namely teacher-prescribed questions (T), Instructional material-guided questions (I), and student-discovered questions (S). Among them, teacher-prescribed questions are the most traditional model; they not only assess students’ understanding, consolidate factual knowledge, and elicit prior knowledge, but also stimulate students’ thinking and promote classroom interaction and student engagement (Peen and Arshad, 2014). As the structure of teaching and learning has shifted and students have become more prominent as subjects of learning, student-initiated questioning has become a common way of teaching and learning, and research has shown that student-initiated questioning facilitates the revelation of students’ thinking, their understanding of concepts, and what they want to know. At the same time, it is also conducive to stimulating students’ subjective consciousness and tapping their learning potential (Guo, 2013). However, although it is highly educational for students to discover problems on their own, in reality, only a few students spontaneously ask advanced or open-ended questions. Therefore, many researchers have conducted in-depth investigations on how to guide students’ problem discovery, e.g., Almeida (2012) suggested that visualization through mind mapping can help students ask questions. In addition, Liu (2017) summarized 10 teaching strategies to promote students’ discovery of problems to ask questions. Teachers’ guidance of students’ discovery of problems by providing relevant learning resources, constructing learning situations, and guiding students to discover problems is also an important source of semi-structured questions.

##### 2.2.1.2 Application of problem-solving scaffolding

Scaffolding stems from Vygotsky’s theoretical view of constructivism, and pedagogical scaffolding can also be referred to as scaffolding. Problem-solving scaffolding is related to the problem-solving process, which is the problem prompts and process guidance provided by the teacher in organizing students to engage in creative problem-solving. In general, some researchers have fully adopted the existing creative problem-solving process model as a scaffold for instructional process guidance to help students follow common creative problem-solving processes for learning, such as discovering problems, discovering ideas, and discovering problem solutions. For example, Cao et al. (2015) provided students with creative problem-solving process scaffolds such as problem prompts, refinement prompts, reflection prompts, and strategy prompts, revealing the positive effects of scaffolded creative problem-solving on students’ learning satisfaction and self-efficacy.

##### 2.2.1.3 Application of thinking tools

Thinking tools are common teaching tools in creative problem-solving. Different from problem solving scaffolds that are used throughout the problem-solving process and guide students’ learning step-by-step (e.g., design thinking five stage model, double-diamond model, etc.); thinking tools tend to be applied at some stage of the problem-solving process to facilitate learner’s thinking activities (e.g., brainstorming, six thinking hats, SCAMPER, empathy map, etc.). These tools can help learners to visualize the thinking process, facilitate ideas exchange, self-reflection, and ultimately improve the divergence and convergence of thinking. For example, Almeida (2012) examined the effectiveness of brainstorming strategies in fostering creativity through a quasi-experimental study, and Wu and Wu (2020) introduced SCAMPER into information engineering project-based learning, and examined its effects on students’ cognitive and motivational aspects. However, the effectiveness of thinking tools is often limited by the differences in practitioners’ experiences, and their actual use may differ from the original design intention; therefore, this study takes the application of thinking tools into consideration in order to clarify the actual impact of the application or non-application of thinking tools on the effectiveness of creative problem-solving.

##### 2.2.1.4 Grouping method

Collaborative learning is one of the main teaching and learning styles nowadays, and grouping, as a necessary part of collaborative learning, plays a crucial and influential role in the construction of student groups (Baer, 2003). As research continues, grouping patterns have become increasingly diverse and complex, ranging from random grouping to grouping based on specific criteria. Currently, the most common grouping patterns are teacher-assigned groups (random or systematic assignment, homogeneous or heterogeneous) and student-chosen groups (Nhan and Nhan, 2019). Homogeneous grouping refers to grouping students based on characteristics such as ability, gender, or ethnicity, ensuring that the same group of students has the same characteristics. Heterogeneous grouping, on the other hand, is the opposite of homogeneous grouping; it involves combining students who possess different characteristics to create balanced teams (Van Der Laan Smith and Spindle, 2007). Random grouping is the most traditional type of grouping; it combines learners together randomly. Student-directed grouping is one of the most favored ways in which students become more familiar with each other in such groups, enabling them to communicate more easily (Swanson et al., 1998).

As can be seen, randomized grouping, student-directed grouping, heterogeneous grouping, and homogeneous grouping are more established and widely used grouping styles, and in the past, although some researchers have compared the effects of homogeneous and heterogeneous grouping on students’ creativity in two-person groups (Triandis et al., 1965), there is still a lack of research comparing the strengths and weaknesses of the four relatively dominant grouping styles in a complete way.

*Application of technology.* We distinguish between studies in which IT tools were used in the problem-solving process and those in which they were not used. IT intervention, also called IT integration, refers to allowing IT to contribute positively to the performance of complex information systems, and it encompasses the formal and informal use of IT by students and teachers inside and outside the classroom (Viberg et al., 2023). As information technology (IT) education continues to grow, more and more countries are enacting legislation and action plans to promote the integration of IT and education, and researchers are increasingly concerned about the impact of IT interventions on students. While the ultimate goal of IT intervention is to cultivate students’ innovative spirit and practical ability (Sun, 2004), an important research direction is the impact of IT intervention on students’ creativity (Yalcinalp and Avci, 2019). Currently, many studies have focused on the effects of different information technologies on students’ creativity at different grade levels (Yalcinalp and Avci, 2019), and these important findings have not yet been integrated into a systematic conclusion. Therefore, a summarized review of the literature on the impact of IT interventions in teaching and learning on creativity can better provide educators with input on accessing IT for teaching and learning.

In order to ensure the reliability and validity of coding, the coding form was completed by two independent researchers in this study, and after the first coding, the kappa consistency test for each subgroup was within the range of 0.6 to 0.8. In order to improve the coding consistency, the two researchers discussed and reworked the papers and corresponding data where inconsistencies existed, and the final kappa values obtained were all greater than 0.8, indicating high internal consistency of the coding results.

#### 2.2.2 Effect size

Effect sizes were used to indicate differences in creativity between PSP instruction and traditional instruction. In order to make the results of the analyzes comparable across studies, standardized effect sizes, or standardized mean differences (SMDs), were calculated for continuous variables in this study using R software. SMD was used as a summary statistic in the meta-analysis when all studies assessed the same outcome but measured it in different ways. In the included studies, the researchers chose different methods to assess students’ creativity, which is consistent with the context in which SMD is applied. Taking into account the bias caused by different sample sizes, Hedge’s g was reported as the effect size, which can be obtained by calculating the mean scores, standard deviations, and sample size data provided by pre-test-post-test experimental or quasi-experimental studies.

### 2.3 Data analysis

#### 2.3.1 Selection of models

Mixed effects models were used in this meta-analysis. A mixed effects model is a combined model that uses a fixed model and a random model to assess differences in between- and within-group effect sizes at the subject level, respectively, and takes into account context-specific variables other than random variables. The mixed-effects model was more appropriate for this study due to the variety of instructional designs and the different backgrounds of the subjects, as well as other factors. The study was prearranged with several moderating variables to consider the influence of potential factors on the overall effect size.

The study used Cochran’s Q statistic (Cohen, 1992) and the I^2^ index to evaluate between- and within-group heterogeneity. The total Q-statistic can be further divided into Q-between and Q-within, which are used to indicate between-group differences and within-group differences. Significant q-statistics and higher I^2^indicate that the effect size comes from different groups (heterogeneity).

#### 2.3.2 Publication bias

Publication bias is the fact that statistically significant findings are more likely to be reported and published than insignificant and invalid findings. In this study, non-parametric methods such as funnel plot, Egger’s test and the trim-and-fill method were firstly used to estimate the possible bias. In the funnel plot, symmetrical numbers indicate no publication bias, and asymmetrical numbers indicate potential publication bias. For the Egger test, the presence of publication bias was determined by the effect of the regression effect size on the precision measure. A regression intercept close to zero indicates no publication bias. The trim-and-fill approach attempted to recover studies lost due to publication bias and to re-estimate the overall effect, assuming that the missing studies were added to the analysis.

**Figure 2:**
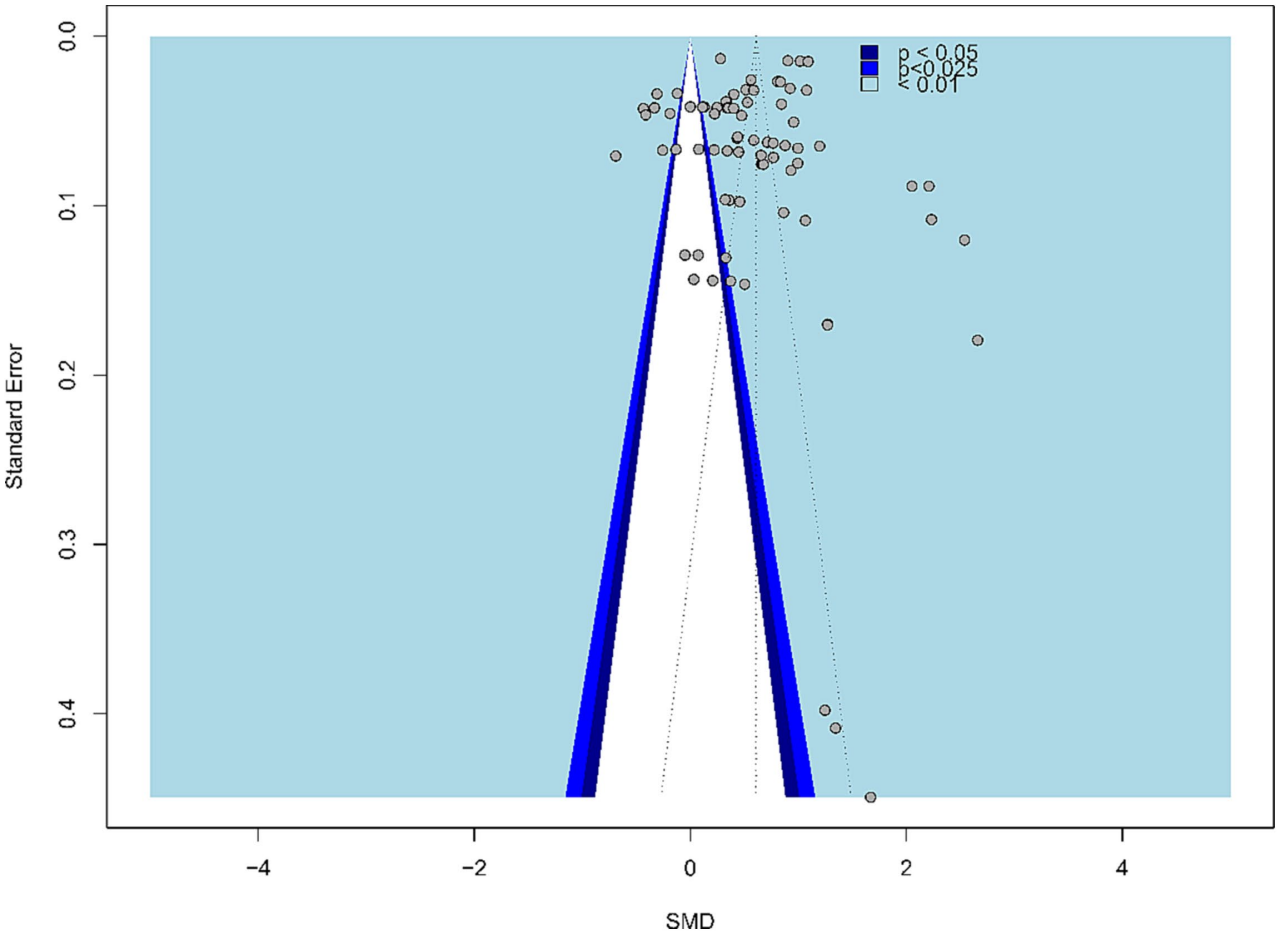
Funnel plot of the random-effect model.

To assess publication bias, we first created a funnel plot, as shown in Figure 2. Intuitively, the funnel plot shows asymmetry, indicating that potential studies may be missing on the left side. As unpublished studies are more difficult to obtain, this study only obtained a small amount of gray literature from ResearchGate and Google Scholar, which may be the reason for the asymmetry of the funnel plot.

**Figure 3:**
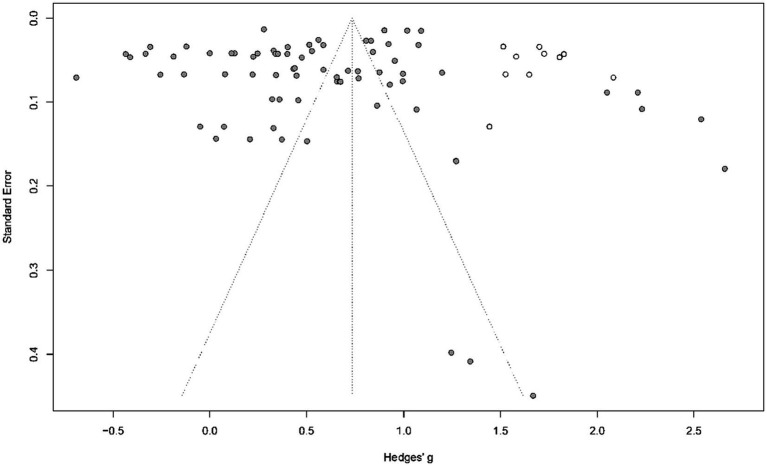
Funnel plot of the random-effect model after Trim-and-Fill.

The study conducted an Egger’s test to statistically argue for the possible meaning of the funnel plot. The results of the Egger’s test showed that publication bias did not have a significant effect on the overall effect (*t* = −1.202, *p* > 0.05), suggesting that no publication bias effect was found. In addition, this study used the tinkering method (Trim and Fill) to explore the number of potential studies that were not included, and the forest plot after tinkering is shown in Figure 3. The results of the Trim and Fill method predicted that potentially 10 studies were not included, and the total effect estimate of the Trim and Fill was SMD = 0.74, 95% CI [0.59, 0.89], *p* < 0.001, which further affirmed the current conclusions, although heterogeneity remained high (I^2^ = 99.4%, *p* < 0.001). As the patch method is an idealized effect size recommendation, overall, the publication bias profile of this meta-analysis was good.

#### 2.3.3 Sensitivity analysis and impact analysis

Sensitivity analysis and impact analysis are methods used to explore inter-study heterogeneity and to help researchers find outliers and test the robustness of the overall effect value.

Sensitivity analysis is a method to recalculate the overall effect value by excluding detected outliers to determine whether the results are subverted and heterogeneity is dissipated. Impact analysis is a method of finding the most valuable studies that have the greatest impact on the overall effect by excluding single studies, measuring the overall effect again, and comparing it to the original overall effect. In the R language package for impact analysis, each study can be eliminated once to complete a round of analysis. This means that impact analysis helps the researcher to identify particular studies that have a significant impact on the overall effect for more in-depth analysis.

#### 2.3.4 Subgroup analysis

The final step of this meta-analysis was to conduct subgroup analysis, for which seven subgroups were created, including sub-variables, instructional method, grade level, grouping method, application of problem-solving scaffolding, sources of problems, application of information technology, and application of thinking tools. In the subgroup analysis, the study calculated the heterogeneity between the different subgroups and calculated the effect size, confidence intervals, and within-group heterogeneity for each group with a view to clarifying the sources of heterogeneity in the overall effect.

## 3 Results

### 3.1 Overall effect of problem-solving instruction on creativity

A total of 19 studies were included in the meta-analysis of this study, resulting in a total of 77 effect sizes, which were derived from independent studies that measured multiple relevant outcome variables, such as creative works, creativity, and thinking fluency; thus, these studies provided multiple effect sizes. To explore the overall effect of PSP on creativity, this study conducted separate overall effect size calculations based on a fixed-effects model and a random-effects model, which are shown in Table 3. For the fixed-effects model, SMD = 0.60, 95% CI [0.59, 0.61], *p* < 0.001; for the random-effects model, SMD = 0.61, 95% CI [0.46, 0.76], *p* < 0.001, both at a statistically significant level. According to the statistical interval of effect sizes proposed by Cohen (1992), a moderate effect is considered when the effect size is in the range of 0.2 to 0.5, and a significant effect is considered when the effect size is ≥0.8; therefore, in this study, teaching creative problem-solving had a moderately positive effect on the development of creativity.

**Table 3:** Summary of the effect sizes obtained in fixed and random-effect meta-analyzes.

	*k*	Effect (SMD)	95% CI LL	95% CI UL	*p*	Q
FEM	77	0.60	0.59	0.61	0.000	9678.40
REM	77	0.61	0.46	0.76	0.000	9678.40

*k*, the number of studies; FEM, Fixed-effects model; REM, Random-effects model; 95% CI, confidence interval; LL, lower limit; UL, upper limit.

*** p < 0.001.

**Figure 4:**
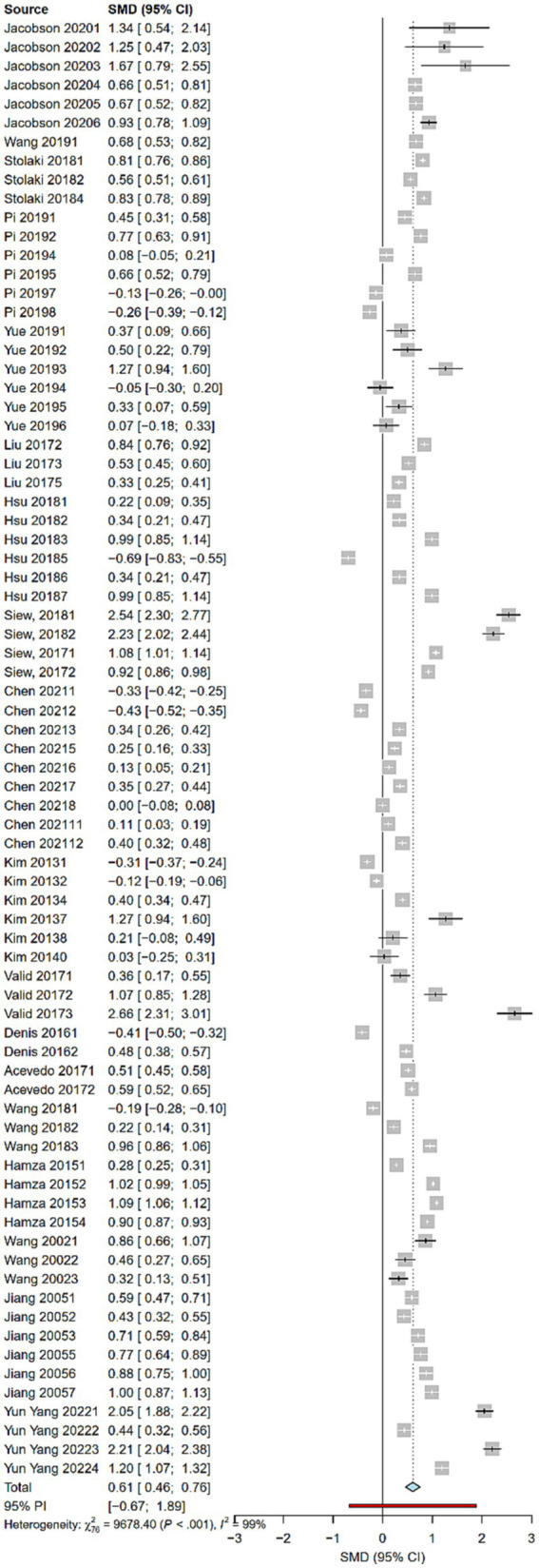
Forest plot of the random-effect model.

To visualize the results more, we plotted a forest plot covering all the included studies, and presented information on individual study effect sizes, confidence intervals, and heterogeneity, as shown in Figure 4. In terms of heterogeneity (Q = 9678.40, *p* < 0.001) and (I^2^ = 99.2%, *p* < 0.001). The data indicated significant differences in effect sizes between studies, meaning that there was a great deal of heterogeneity across studies. Therefore, further examination of heterogeneity is warranted. In order to explore the causes or sources of heterogeneity, subgroup analyzes were conducted in this study for different moderator variables.

### 3.2 Sensitivity analysis and impact analysis

Outliers were identified by comparing the overlap between the confidence intervals of the individual studies and the confidence intervals of the combined effect. In this study, a total of 23 outliers were identified, and after removing the outliers, the meta-analysis was re-run on the remaining 54 data and found that the new combined effect was SMD = 0.31, 95% CI = [0.21, 0.42], which was smaller than the original combined effect but statistically significant (*p* < 0.01). The original conclusions of the present study were thus consolidated. However, as the heterogeneity remained large after excluding outliers (I^2^ = 98.7%, *p* < 0.001), suggesting that these outliers were not the cause of the heterogeneity, this study retained this portion of the data in subsequent analyzes. In addition, the results of the impact analysis did not identify influential studies with significant impacts, suggesting that the combined effect sizes of this study are not dependent on a specific single study and are robust.

### 3.3 Subgroup analysis

Based on the mixed-effects model, Table 4 summarizes the effects of multiple prespecified moderating variables on the overall effect of creativity in PSP, specifically including information on between-group heterogeneity, within-group heterogeneity, subgroup effect sizes, and 95% confidence intervals.

**Table 4:** Effect sizes for all coded variables.

Moderator variables	*n*	Effect size	*p*	Mixed-effects model				
				95%CI		Qw	Qb	P_Heterogeneity_
		SMD		Lower	Upper			
*Creativity predictor*							5.72	0.057
Fluency	28	0.55	0.000	0.26	0.84	3672.57		
Flexibility	19	0.43	0.000	0.26	0.61	2170.93		
Originality	30	0.77	0.000	0.55	0.99	2180.58		
*Instructional method*							0.62	0.432
Collaborative creative problem-solving (CCPS)	53	0.65	0.000	0.51	0.80	4912.60		
Individual creative problem-solving (ICPS)	24	0.51	0.003	0.18	0.84	2509.28		
*Grouping method*							10.87	0.028
Heterogeneous grouping (HeG)	4	1.69	0.000	0.89	2.48	285.32		
Homogeneous grouping (HoG)	6	0.26	0.134	−0.08	0.60	190.50		
Random grouping (RG)	19	0.56	0.000	0.37	0.75	3079.22		
Students grouping (SG)	9	0.52	0.000	0.26	0.78	153.32		
*Grade level*							22.92	0.000
Elementary school (E)	11	1.20	0.000	0.70	1.70	567.24		
Middle school (M)	8	0.31	0.068	−0.02	0.65	629.38		
Higher education (H)	18	0.23	0.016	0.04	0.42	782.35		
University (U)	31	0.55	0.000	0.38	0.73	4172.38		
Other (O)	9	1.06	0.000	0.62	1.50	519.81		
*Problem-solving scaffolding*							0.00	0.969
No	33	0.61	0.000	0.41	0.81	4215.69		
Yes	44	0.61	0.000	0.41	0.82	4087.80		
*Application of technology*							0.46	0.498
No	51	0.65	0.000	0.47	0.82	6747.24		
Yes	26	0.54	0.000	0.28	0.80	2710.55		
*Sources of problem*							17.14	0.000
Instructed by guided materials (I)	13	0.24	0.001	0.05	0.44	829.37		
Student-discovered questions (S)	15	0.70	0.000	0.60	0.80	152.23		
Teacher-prescribed questions (T)	49	0.68	0.000	0.47	0.90	7517.55		
*Application of thinking tools*							3.70	0.054
No	24	0.43	0.000	0.25	0.62	2082.23		
Yes	53	0.69	0.000	0.50	0.88	7523.76		

#### 3.3.1 Creativity secondary dimensions

This study subdivided the secondary variables of fluency, flexibility, and originality, which are more representative of creativity, to explore whether PSP has a focused impact on different manifestations of creativity. The resultant data from this subgroup analysis indicated no significant between-group heterogeneity (Qb = 0.06, *p* > 0.05), meaning that there was no significant difference in the effect of creative problem-solving pedagogy on students’ performance on fluency, flexibility, and originality, and that it had a significant positive effect on all three. Although, in terms of the relative values of the effect sizes, creative problem-solving instruction was more conducive to promoting student originality (SMD = 0.77, *p* < 0.001).

#### 3.3.2 Instructional method

According to the different instructional methods, scholars’ research on PSP can be specifically categorized into individual problem-solving and collaborative problem-solving. This meta-analysis was conducted to analyze the subgroup with this teaching style as the moderating variable, and the data showed that the between-group heterogeneity of this subgroup was not significant (Qb = 0.62, *p* > 0.05), i.e., there was no significant difference between the effects of the two teaching styles of Individual Problem-solving (SMD = 0.51, *p* < 0.001) and Collaborative Problem-solving (SMD = 0.65, *p* < 0.001) on the development of creativity, both of which had a positive impact on creativity. Relatively speaking, collaborative problem-solving instruction may be more favorable for developing students’ creativity.

#### 3.3.3 Grouping method

In order to determine the influence of grouping styles on the overall effect size in PSP, the study used grouping style as a moderating variable and divided it into subgroups of heterogeneous grouping, homogeneous grouping, randomized grouping, and free grouping of students. The results of the between-group heterogeneity test for this subgroup demonstrated statistical significance (Qb = 10.87, *p* < 0.05). It was found that, similar to previous studies, collaborative creative problem-solving under heterogeneous subgrouping was more effective in fostering creativity (SMD = 1.69, *p* < 0.001), followed by randomized subgrouping (SMD = 0.56, *p* < 0.001) and students’ free grouping (SMD = 0.52, *p* < 0.001). Students’ collaborative creative problem-solving performance was the poorest in the homogeneous subgrouping (SMD = 0.26, *p* > 0.05).

#### 3.3.4 Grade level

As seen from the included empirical studies, problem-solving instruction is widely used at different school ages, so this study took into account the variability of learners at different stages, and subgroup analysis of the application of creative problem-solving at different school stages, which can be categorized into elementary, middle, high school, university, and on-the-job, was conducted using school stage as a moderating variable. The results of the between-group heterogeneity test for this subgroup indicated a statistically significant difference between the groups (Qb = 22.92, *p* < 0.001), as evidenced by the fact that the best results were obtained for instruction geared toward the elementary school level (SMD = 1.20, *p* < 0.001), followed by the in-service level (SMD = 1.06, *p* < 0.001), the university (SMD = 0.55, *p* < 0.001), and the secondary level (SMD = 0.31, *p* > 0.05), with the least significant effect of creative problem-solving instruction geared toward high school level learners (SMD = 0.23, *p* < 0.05).

#### 3.3.5 Application of problem-solving scaffolding

As scholars have different understandings of creative problem-solving, some scholars directly adopted the common creative problem-solving process model to organize teaching, while others guided students to diverge and converge their thinking in the process of problem-solving in their teaching concepts or teaching tools to ultimately achieve creative problem-solving. Therefore, this study conducted a subgroup analysis using the application of problem-solving scaffolds as a moderating variable, distinguishing between studies that used problem-solving scaffolds and those that did not. Between-group heterogeneity in this subgroup did not reflect statistically significant differences (Qb = 0.001, *p* > 0.05). There was no significant difference between instruction with the application of problem-solving scaffolding (SMD = 0.61, *p* < 0.001) and instruction without the application of problem-solving scaffolding (SMD = 0.61, *p* < 0.001) in terms of the effectiveness of creativity development.

#### 3.3.6 Problem source

Based on the moderating variable of problem source, three types of problems were classified as teacher-prescribed questions (T), Instructional material-guided questions (I), and student-discovered questions (S). The between-group heterogeneity of this subgroup reflected a statistically significant difference (Qb = 17.14, *p* < 0.001). In terms of the PSP effect on creativity, student-discovered questions were most conducive (SMD = 0.70, *p* < 0.001), followed by teacher-prescribed questions (SMD = 0.68, *p* < 0.001), while Instructional material-guided questions were less effective in promoting creativity (SMD = 0.24, *p* < 0.05).

#### 3.3.7 Application of thinking tools

Based on the application or non-application of thinking tools, the dataset can be divided into two subgroups to analyze the differences in the effects on students’ creativity between PSP with the application of thinking tools and that without. The results of between-group heterogeneity pointed out that the difference in the effect on creativity development between the application of thinking tools and the non-application of thinking tools did not meet the requirement of statistical significance (Qb = 0.054, *p* > 0.05), and that, comparatively speaking, the PSP with the application of thinking tools was more effective (SMD = 0.69, *p* < 0.001).

#### 3.3.8 Application of technology

Depending on whether information technology was applied or not, the dataset could be divided into two subgroups to analyze the effect of information technology on students’ creativity under PSP. The results of heterogeneity between the groups pointed out that the difference in the effect on creativity development between the application of information technology and the non-application of information technology did not meet the requirement of statistical significance (Qb = 0.46, *p* > 0.05), and that, relatively speaking, PSP without the application of information technology was more effective in fostering creativity (SMD = 0.66, *p* < 0.05).

## 4 Discussion

In this study, we examined the effectiveness of PSP on students’ creativity development in a meta-analytic manner. After an extensive search, 19 relevant educational pilot studies were included in the database, reporting a total of 77 usable descriptive statistics. However, the strength of the effectiveness of PSP on student creativity varies across moderating variables. Specifically, we summarize the following findings.

### 4.1 Creativity overall effect

Regarding RQ1, the results of the meta-analysis indicated that classrooms with PSP were more conducive to the development of student creativity than traditional methods. It is probably because PSP allows students to fully experience the problem-solving process. Puccio and Cabra (2010) pointed out that the process of innovation is the thinking process that people go through when they creatively cope with the difficulties and opportunities in work alone or in cooperation with others, and the creative effort often does not end until the product of creative thinking is formally completed. The tasks under the guidance of PSP are often divided into multiple links (Klegeris et al., 2013), which requires learners to step-by-step propose complete and feasible solutions to problems, which is more conducive to the improvement of learners’ creativity than conventional PBL.

### 4.2 Creativity secondary dimensions

Regarding RQ2, since previous studies have often expressed levels of creativity in terms of fluency, flexibility, and novelty, the present study further examined the effects of PSP on the different elements of creativity. It is found that PSP has positive effects on all the three different dimensions of creativity, which is consistent with previous studies (Wang, 2019; Probowati et al., 2020). Specifically, originality is the most affected among the three dimensions, which may be due to the fact that in PSP-guided teaching, teachers often expose students to a large number of creative examples and encourage them to produce novel ideas, so students gain greater creative originality (Wang, 2019).

### 4.3 Instructional method

Regarding RQ3, the difference between the two instructional methods, individual and collaborative learning, on students’ creativity was not significant, while the impact of collaborative creative problem-solving on creativity development was relatively more effective. On the one hand, this may be due to the large gap in the number of studies reported on collaborative learning (*n* = 53) versus individual learning (*n* = 24). On the other hand, considering that the rich exchange of discussions in collaborative learning is more conducive to realizing the accumulation of the number of ideas and the diffusion of the range of ideas than individual learning, probabilistically increasing the likelihood of quantitative to qualitative epiphanies occurring (Palmgren-Neuvonen and Korkeamäki, 2014), and especially in the context of attempting to create innovations, students have a greater need to capitalize on the synergies between internal and external sources of creativity at the individual level and at the collaborative level (Steiner, 2009).

### 4.4 Grouping method

Regarding RQ4, research in the field of organizational innovation has thoroughly explored how to organize groups for collaborative learning. The complex attributes of creative teams, such as team composition, organizational strategy, and work norms, may all have an impact on creativity performance (Cirella et al., 2014). Collaboration under different grouping styles had different effects on creativity performance, with heterogeneous grouping styles having the most prominent positive effect, randomized grouping styles being in the middle of the list, and homogeneous grouping styles having the least significant effect. This is similar to the idea that team diversity contributes to creativity as mentioned by Manukyan et al. (2013) and Zhan et al. (2015, 2022a,b). In addition, the creativity performance of collaborative creative problem-solving under randomized grouping and free teaming of students was in the middle of the positive effect. In the included study, the freely formed groups of students tended to be interest-oriented, i.e., each person came together based on his or her level of interest in a problem. Such groups would be more intrinsically motivated for problem-solving, and their creativity performance may be better (Fischer et al., 2019). Randomized grouping, on the other hand, although less diverse than heterogeneous grouping in terms of team diversity, can also help the group to some extent to gain a greater sampling of perspectives in the collaborative process, which, in turn, has a better impact on creativity than homogeneous grouping.

### 4.5 Grade level

Regarding RQ5, in terms of the effect of PSP use on creativity in different grade levels, the five main grade levels in the current study include elementary school, middle school, high school, college, and in-service. The best results of PSP use were found in elementary school, which may be due to the fact that elementary school learners are younger, and their thinking is more likely to be influenced by PSP as they have not yet developed stereotypes (Balasanova et al., 2020), or their socialization and herd mentality has not yet been developed (Kim, 2011). This has been verified in past studies such as Gardner and Gardner (2008), who noted that preschoolers have a high level of creativity, but when they enter school their creativity declines as they learn to conform. In contrast, the reason why high school and middle school students are not achieving good results in creativity could be from the influence of academic pressure. Muirhead (2011) showed that when students are performance-oriented in their learning, it makes the need to enjoy the learning process unfulfilled, which in turn leads to the PSP not being able to deliver its full value. Smith and Carlsson (1983) also noted an increase in obsessive and obsessive-like strategies among learners at the middle school level, which leads to difficulties in enhancing creativity.

### 4.6 Application of problem-solving scaffolding

Regarding RQ6, the unexpected finding was that the use of the traditional scaffolding of the problem-solving process and the non-use of scaffolding had the same effect on creativity development. Although numerous studies on PSP have claimed that the application of their proposed scaffolds is significantly important for creativity development, in terms of the results of the present study, whether or not the model is applied may not bring about an intrinsic effect on the effectiveness of creativity development. This may be due to the fact that many studies have directly applied the validated problem-solving scaffolds in the use of PSP, and failed to make appropriate modifications according to the content of the instruction and the characteristics of the learners. This may have led to a much lower impact of PSP on students’ creativity. Past research has also indicated that domain-specific scaffolding has a more pronounced impact on students than general domain scaffolding demonstrations (Bulu and Pedersen, 2010). In addition, as Amabile (2011), a proponent of creativity achievement theory, argues, creativity development is the overall result of the elements of a complex educational environment, and scaffolding is only one of the elements, while other elements such as students’ personality, affective attitudes, knowledge base, and classroom climate are also influential. This suggests that when teaching creative problem-solving, we should not simply apply creative problem-solving scaffolding to the instructional design, but should consider a variety of variables that affect the development of creativity.

### 4.7 Problem source

Regarding RQ7, this study found significant differences in the effects of different sources of questions on creativity, with student-discovered questions being the most conducive to creativity stimulation, followed by teachers prescribed questions, and Instructional materials-guided questions being less effective in terms of promoting creativity. A research finding that echoes the theory of creativity and organizational innovation lies in the fact that students’ spontaneous questions are more conducive to their creativity (Amabile, 1988). At the individual level, creativity is influenced by intrinsic motivation and interest in the task (Moirano et al., 2020), and creative problem-solving activities are more favorable when students pose problems of interest, driven by intrinsic motivation. Compared to students’ free problem formulation and teacher-proposed problems, material-based problem discovery is a semi-structured source of problems, and this meta-analysis led to the more negative conclusion that guiding students’ problem discovery through materials has little effect on the PSP for creativity development. Zheng and Yin (2012) suggested that, in the midst of semi-structured problem-solving instruction, students are more likely to be motivated and interested in problem-solving activities due to a lack of metacognitive skills. Students seem to have difficulty identifying a specific problem and developing creative solutions, and the intervention of problem prompts can improve this pedagogical problem by guiding students to make conscious and meaningful efforts to define and analyze the problem.

### 4.8 Application of thinking tools

Regarding RQ8, an unexpected finding was that there is no significant difference in the development of creativity between PSP that applies thinking tools and instruction that does not emphasize the application of thinking tools. In the history of creativity research, many studies have developed divergent thinking tools such as Brainstorming, Six Thinking Hats, and SCAMPER, and have argued for the effectiveness of these tools in terms of creativity development (Hsu et al., 2018; Jacobson et al., 2020). In the studies included in this meta-analysis, thinking tools were mostly applied in team situations, but the complex social nature of group participation may have an inhibitory effect on creativity (Reiter-Palmon et al., 2012). On the other hand, when teachers use the aforementioned thinking tools, they often reduce them to prescribed steps of a certain kind of thinking. As creativity research deepens, a growing number of scholars are arguing that the promotion of creativity should focus more on non-linear processes, and that prescriptive approaches should shift to descriptive approaches (Sawyer, 2021).

### 4.9 Application of technology

Regarding RQ9, this study found that there is no significant difference between the effect of IT use and non-use on creativity development; instead, the PSP without the use of IT was more effective in terms of effect size values. This may be related to the way IT is applied. It has been suggested that brainstorming tools that support online collaboration are more conducive to idea elicitation than traditional face-to-face brainstorming tools, avoiding the loss of ideas while waiting to speak (Korde and Paulus, 2017). Similar collaborative communication tools may lead to similar effects. However, in some of the included studies, some scholars only used IT as a tool for resource sharing, and other studies used specific learning systems. Therefore, it is necessary for future research to examine the effects of IT on creativity by analyzing subgroups for differences in the way IT is applied. In addition, studies of IT applications in teaching and learning tend to consider the effects of technology acceptance, and poor student technology acceptance and proficiency may curb learning outcomes (Buche et al., 2007). On the other hand, media richness may have a negative impact on creativity (Runco, 2015) and the significant difference in the effectiveness of communication in virtual spaces versus face-to-face communication may lead to lower levels of learner perceptions of the importance of group membership and belonging (Mckinlay et al., 1999). In addition, when we communicate in virtual space, it can lead to difficulties in observing the nuances of each other’s facial expressions and body language, which in turn may reduce our understanding of each other’s perspectives (Hilliges et al., 2007). However, the included studies largely failed to report on the specifics of students’ use of information technology, i.e., there is a lack of existing experimental studies reporting on more pedagogical details or of guidance on students’ technology use, which resulted in students’ failure to use the technology correctly (Stolaki and Economides, 2018; Wang et al., 2019). Referring technology application (RQ9) and instructional method (RQ3), it is fount that technology adopted in team-based learning is more effective than that used individually.

Referring the results of technology Application (RQ9) and Grade level(RQ5), the results of this study demonstrated that the PSP without using of information technology has a better effect. This finding is out of our expectation. As previous studies indicated that the impact of digital technology on students’ creativity depends on teaching strategies according to students’ learning status (Tang et al., 2022), it is supposed that the teachers fail to select appropriate technology and apply appropriate teaching strategies according to students’ characteristics and level of cognition. For the elementary school stage, considering the students’ low acceptance of technology, information technology has not greatly influenced the effect of PSP on creativity. Whereas, for university or post-service education stage, learners have the highest acceptance of technology and distance learning is prevalent, so that PSP on creativity in these two grade levels are widely recommended. While for the stage of middle and high school, students have greater pressure on examination, and the intervention of technology on PSP may cause their resistance. This may be the reason why the PSP’s effect on creativity is not significant overall.

Referring the results of problem-solving scaffolding application (RQ6), problem source (RQ7), and thinking tool application (RQ8), this study found that students’ spontaneous questions are most conducive to creativity stimulation, while guided discovery questions has a less significant effect. Generally, the first step in problem-solving scaffolding is to ask the learner to ask the question. In order to ensure that the questions raised by students fit the teaching content, teachers often give guidance to students, which may weaken the autonomy of learners, resulting in the influence of PSP on students’ creativity in problem-solving. In addition, some teachers provides students with thinking tools (e.g., visualization through mind mapping) within the scaffolds, which showed better effect on promoting the impact of PSP on creativity (Stokhof et al., 2020).

Referring the results of instructional method (RQ3) and problem-solving scaffolding application (RQ6), this study found no significant difference on both moderators. As it is known, better group performance does not necessarily mean better individual performance, and vice versa. One plausible explanation for this is the different requirements for group performance and individual performance. The former reflects the collective wisdom and efforts from all group members, while the latter requires each member of the group to actively participate and fully interact (Woolley et al., 2010). Therefore, when the problem-solving scaffolding used does not guide students how to collaborate, the interaction contradictions and corresponding cognitive load brought about by collaboration may weaken the advantages brought by collaborative problem solving, that is, collaborative problem solving may not necessarily achieve better results than individual problem solving (Lou et al., 2001).

## 5 Conclusion

This study explored the relationship between PSP and the development of students’ individual creativity under different teaching contexts and conditions, and summarized seven different teaching conditions and contexts based on existing research: Grade Level, Instructional Method, Grouping Method, Sources of Problem, Problem-Solving, The Application of Scaffolding, The Application of Thinking Tools and The Application of Technology. It analyzed the effects of PSP on students’ individual creativity under the support of these conditions. The results of this study show that, in general, PSP has a positive effect on students’ creativity, and has a consistent effect on all elements of creativity, i.e., PSP can promote the overall enhancement of creativity. However, the effect of its influence changes under particular conditions, and among the conditions included in this study, the instructional conditions that had a significant effect on students’ creativity included the source of the problem, the grouping method, and the learners’ academic period. All of these effects essentially stem from their impact on students’ thinking during the creative problem-solving process, which includes the motivation to think, the richness of the available ideas, and the ease of change in the students’ thinking itself. For example, the student-initiated questions in the problem sources increased students’ interest and enabled them to think more actively, and the heterogeneous groupings in the grouping styles enabled learners to gain richer perspectives and to be inspired more easily. The elementary school stage is the stage where the learners’ thinking is not regulated, and the learners’ thinking is more active and easier to be improved. However, the present study did not reveal significant benefits for creativity enhancement in the four conditions of Instructional Method, Problem-Solving Scaffolding, Application of Thinking Tools and Application of Technology. This is not in agreement with many studies, and the reason for these phenomena can be attributed to the lack of flexibility in the use of instructional tools in conjunction with the characteristics of creativity. Creativity consists of three main elements: fluency, flexibility and originality; flow requires learners to be familiar with the use of learner tools; flexibility requires that the process of its cultivation is a non-linear process, and linear scaffolding is not suitable for the process of creative problem-solving. Originality requires not only sufficient personal reflection, but also a sufficiently intense collision of minds to obtain new ideas between the intermixing of different minds. Therefore, when applying these teaching tools, it is necessary to consider the needs of students’ personal inquiry and the needs of cooperative communication, i.e., to give students access to diversified viewpoints, but also to avoid the burden of too many viewpoints on students’ personal thinking.

## 6 Limitations and future study

This study developed a meta-analysis of the pedagogical elements surrounding the teaching of creative problem-solving to summarize the current practical effects of creative problem-solving teaching on creativity development, and to provide inspiration for how to design teaching for creativity development. However, this study could be improved as only a limited number of studies were included. There is a need to expand the database search and add missing literature in future studies to avoid biased conclusions. In addition, the limited number of studies correspondingly restricted the depth of subgroup analysis. Future studies could look at the ability to apply information technology, the basis for heterogeneous grouping, and the application of different thinking tools.

In terms of information technology application, this study explored the effects of information technology-supported PSP on learners’ creativity; however, while exploring a variety of approaches to information technology application and creativity development, this study did not explore students’ technological proficiency in depth. Past research has shown that information technology plays an integral role in the creative process, and that students’ technological proficiency affects the effectiveness of information technology use. Future research could further explore what kind of information technology is more appropriate to be applied to learners with different technological proficiencies in order to support the development of creativity.

In terms of grouping, this study found that heterogeneous grouping has the greatest impact on the development of students’ creativity; however, due to the limitations of the number of studies, this study did not further explore the basis for heterogeneous grouping in different studies. Future research could further analyze the impact of different heterogeneous grouping methods on learners’ creativity.

In terms of thinking tools, this study explored the effect of the presence or absence of thinking tools on creativity; however, the method of acting on thinking tools is also an important reason for their effect; future research can explore the stages of acting on thinking tools and application strategies, providing guidance for teachers on how to select and apply thinking tools.

## Data availability statement

The data are available from the corresponding author on reasonable request.

## Author contributions

ZZ: Writing – original draft, Writing – review & editing. LH: Writing – original draft, Writing – review & editing. XZ: Writing – original draft, Writing – review & editing.
